# The Key Indicator Method for Manual Handling Operations (KIM-MHO) - evaluation of a new method for the assessment of working conditions within a cross-sectional study

**DOI:** 10.1186/1471-2474-11-272

**Published:** 2010-11-25

**Authors:** André Klussmann, Ulf Steinberg, Falk Liebers, Hansjürgen Gebhardt, Monika A Rieger

**Affiliations:** 1 Institute of Occupational Health, Safety and Ergonomics (ASER) at the University of Wuppertal, Corneliusstr. 31, 42329 Wuppertal, Germany; 2 Federal Institute for Occupational Safety and Health, Noeldnerstr. 40-42, 10317 Berlin, Germany; 3 Department of Occupational Health and Environmental Medicine, Institute of General Practice and Family Medicine, University of Witten/Herdecke, Alfred-Herrhausen-Str, 50, 58448 Witten, Germany; 4 Institute of Occupational and Social Medicine, University Hospital of Tuebingen, Wilhelmstr. 27, 72074 Tuebingen, Germany

## Abstract

**Background:**

Upper extremity musculoskeletal symptoms and disorders are common in the working population. The economic and social impact of such disorders is considerable. Long-time, dynamic repetitive exposure of the hand-arm system during manual handling operations (MHO) alone or in combination with static and postural effort are recognised as causes of musculoskeletal symptoms and disorders. The assessment of these manual work tasks is crucial to estimate health risks of exposed employees. For these work tasks, a new method for the assessment of the working conditions was developed by the Federal Institute for Occupational Safety and Health (BAuA) and released as a draft in the year 2007. The draft of the so-called Key Indicator Method for Manual Handling Operations (KIM-MHO) was developed in analogy with the existing KIM for Lifting/Holding/Carrying (KIM-LHC) and Pulling/Pushing (KIM-PP) of loads. The KIM-MHO is designed to fill the gap existing in risk assessment of manual work processes, since the existing KIMs deal only with manual handling of loads.

This research project focused on the following:

- Examination of the validity of workplace assessment with the KIM-MHO comparing expert ratings with the results of the observations.

- Examination of the objectivity of workplace assessment with the KIM-MHO applied by different examiners.

- Examination of the criterion validity of the risk assessment provided by KIM-MHO with respect to the association between exposure and the occurrence and prevalence of health related outcomes.

**Methods/Design:**

To determine the objectivity and validity of workplace assessment, the KIM-MHO is applied by occupational health and safety officers at different workplaces involving manual handling operations.

To determine the criterion validity of risk assessment, a survey of employees at different workplaces takes place with standardised questionnaires and interviews about symptoms in the neck and upper extremities. In addition, physical examinations of these employees following a standardised medical diagnostic procedure are also carried out.

**Discussion:**

This research project will provide scientific evaluation of the new KIM-MHO and, if necessary, indicate areas for modification to improve this new method for assessment of the health risk of manual handling operations at diverse workplaces.

## Background

Upper extremity musculoskeletal disorders (UEMSDs) related to work have been recognised for many decades. Upper limb musculoskeletal disorders are still common in the working population [1]. In addition to computer work, heavy loads, high forces, awkward postures, and repetitive movements are the most frequently discussed work-related physical factors [2-6].

According to the European Council Directive 89/391/EEC of 12th June 1989 on the introduction of measures to encourage improvements in the safety and health of workers at work, the employer must perform an assessment of the risks to safety and health at work, including those to which specific groups of workers are exposed [7].

To assess the risk of musculoskeletal symptoms and disorders related to work, a number of assessment methods have been developed. Among others, the "Key Indicator Methods" (KIM) were developed to screen for and assess risks involved in manual handling of loads. Two different KIM Worksheets, one for Lifting, Holding, Carrying of loads (KIM-LHC) and one for Pulling and Pushing of loads (KIM-PP), are already available. These methods were developed by the Federal Institute for Occupational Safety and Health (BAuA) and the Committee of the German states for Occupational Safety and Health (LASI) in close collaboration with practitioners, safety representatives, occupational health physicians, employers and employees associations, insurance organisations, and scientific institutes [8,9]. During the European inspection and communication campaign „LIGHTEN THE LOAD", these methods were translated into all European languages and can be accessed via Internet [10]. Briefly, work characteristics such as posture, load, and frequency are assessed by means of the KIM and a score is calculated to summarise the work-associated risk for musculoskeletal symptoms. To illustrate the results, the score is transformed into a coloured scale indicating a low exposure situation where physical overload is unlikely to occur (= green), situations with increased (= greenish yellow) and highly increased (= yellow) exposure, up to conditions where physical overload is highly likely to occur with probable necessity to redesign the workplace (= red). For a more detailed description please see the section on the KIM-MHO below.

These two KIMs already in use deal only with manual handling of loads, but not with manual handling operations. To fill this gap, a supplemental method was required. Extensive research of scientific and non-scientific literature published predominantly in German and English in 2004 found 37 methods for the assessment of working conditions associated with repetitive hand-arm work/manual work [11-43]. These assessment methods adressed over 150 different individual work characteristics, which could be assigned to 11 main groups: work organisation, breaks, body posture/movement, hand/arm posture, force/load, physiological parameters (e.g. heart rate, blood pressure, recovery time), environmental conditions (e.g. heat, vibration), workplace ergonomics, psychosocial demands, skills, and individual factors. The various methods included on the one hand a large number of work characteristics assumed to be related to health outcomes and - on the other hand - a large number of anatomical regions, symptoms and diseases which could be affected by these work characteristics. There is only little evidence for a specific "cause and effect model" or even a "dose response relationship" between manual handling operations and upper limb disorders. The selection of the observed characteristics varied significantly between the various methods [11-43]. Unfortunately, details about the selection of the parameters were often not indicated in the publications, and many questions remain about the deduction or the combination of the parameters to be assessed. In addition, so far only a small number of instruments or methods assessing biomechanical exposures in occupational settings have been tested in a systematic manner for validity, reliability, objectivity, or other aspects related to their practical application [44].

The development of this KIM-MHO was based on a comprehensive and critical literature review of the methods mentioned above. This knowledge was combined with interviews with scientists, supervisors of state agencies and professional associations, occupational physicians, occupational health and safety officers, and managers from companies in various industries about typical kinds of exposures and exposure-structures of MHO. In 2005, the first draft of the KIM-MHO was field-tested for feasibility at 112 workplaces. At every workplace, the KIM-MHO was completed and discussed with the respective occupational health and safety officers in the companies, and then further developed and improved iteratively. Approximately 20% of the workplaces studied were assigned to the "green" and 10% to the "red" area. In 70% of all cases considered, an increased risk (greenish yellow to yellow) was identified [45]. According to expert discussions after the 112 field-tests, the results seemed to be plausible to the experts involved and consulted. Scientific evaluation of the new KIM-MHO is done, presented and discussed in this study.

## Methods/Design

### Aim of this research project

The aim of this research project is to evaluate the new draft of the KIM-MHO [45,46]. The validity (variance between real exposure and it's assessment by different individuals) and the objectivity (independence of results assessed by different individuals) of workplace assessment with the KIM-MHO are evaluated in this project. A further objective considered is the association between the exposure of manual handling operations as assessed by means of the score of the KIM-MHO and the frequency of musculoskeletal symptoms within the exposed workers (criterion validity). The study is designed as a cross-sectional study in Germany.

### Research topics

The objectives above suggest the following working hypotheses:

1. Assessing workplaces by means of specific instruments by a scientist experienced in ergonomics and by means of occupational health and safety officers using the KIM-MHO, will result in no significant discrepancies.

2. At the completion of the KIM-MHO, no significant discrepancies occur between the assessment by different occupational health and safety officers involved.

3. It is assumed, that employees at workplaces with high exposures of manual handling operations show health related outcomes (musculoskeletal symptoms) more frequently than non-exposed workers, taking into consideration relevant confounders such as age, gender, constitution or disposition. Secondly it is assumed, that the KIM-MHO displays high scores at workplaces with high degrees of manual handling operations and high frequencies of musculoskeletal symptoms in exposed workers, and low scores at workplaces with low exposures of manual handling operations and low frequency of musculoskeletal symptoms in workers.

### Instruments

The instruments (i.e. standardised questionnaire, standardised medical diagnostic procedure, assessment of exposure) used in this survey have been used in a similar form in other studies by several authors. Among them, the authors of the present study applied them for the assessment of musculoskeletal symptoms of upper extremities and the neck in office workers [5]. This former study sample will be used as a reference data set (see Analysis section below).

1) Assessment of health outcome:

- Survey of exposed employees utilising a standardised questionnaire.

- Physical examination of the exposed employees, performing a standardised medical diagnostic procedure.

2) Assessment of exposure:

- Documentation of working conditions by ergonomic work procedure analysis and time analysis including task observation, time measurements, and assessment of technical procedures.

3) Application of the KIM-MHO:

- Application of the KIM-MHO based on the exposure assessment.

#### Standardised questionnaire

The employees' questionnaire is based on the Nordic Questionnaire [47], parts of the Copenhagen Psychosocial Questionnaire (COPSOQ [48]), and the FEBA questionnaire [49]. In our study, the respective German versions of the Nordic Questionnaire [50] and the COPSOQ [51] are applied. The questionnaire contains sociodemographic factors (e.g. age, gender, years on the job, leisure time activities, smoking habits), musculoskeletal symptoms (e.g. prevalence, degree of disability), general working conditions (e.g. time pressure, shift work, working posture), and work-related psychosocial factors (e.g. job satisfaction, cognitive demands, social support).

#### Standardised medical diagnostic procedure

In a SALTSA study, a list of standard diagnoses of musculoskeletal disorders was suggested to analyse the extent to which musculoskeletal symptoms could be attributed to specific tentative diagnoses [52]. The medical diagnostic procedure used in the present study was derived from this SALTSA study. The examination tool consists of a documentation sheet and a reference sheet. The documentation sheet is divided into three parts. Part A is a general survey to document painful or symptomatic body regions. Part B deals with specific examination techniques to be carried out if pain or symptoms in specific regions were documented in part A. According to these results, tentative diagnoses can be assigned using a list of diagnoses in part C. These are:

1. cervical neck syndrome,

2. cervico-brachial neck syndrome,

3. rotator cuff syndrome,

4. medial and lateral epicondylitis,

5. ulnar nerve compression at the elbow: cubital tunnel syndrome,

6. radial nerve compression: radial tunnel syndrome,

7. flexor/extensor peritendinitis/tendosynovitis of forearm/wrist region,

8. de Quervain's disease,

9. carpal tunnel syndrome,

10. ulnar nerve compression at the wrist: Guyon-canal-syndrom,

11. Raynaud's phenomenon (vibration white finger) and peripheral neuropathy associated with hand-arm vibration,

12. osteoarthritis of the distal upper extremities joints, and

13. non-specific upper extremity musculoskeletal disorders (UEMSDs).

As compared to the SALTSA study mentioned above [52] the list of diagnoses was modified for this survey, since usually it is differentiated between cervical neck syndrome and cervico-brachial neck syndrome in Germany. In the original SALTSA study, both were merged to "radiating neck complains".

#### Key Indicator Method - Manual Handling Operations (KIM-MHO)

The new draft of the KIM-MHO is the central topic of this research project. It complements the existing KIMs to assess the working conditions for physical work. In accordance with the principle of the KIMs, it contains an objective requirement and load description, and identifies potential threats to physical overload. The KIM-MHO includes job characteristics and their interaction. The key indicators to be considered in the KIM-MHO are:

• daily duration of manual work processes,

• type, duration, and frequency of executing forces,

• body posture during manual work processes,

• hand-arm posture during manual work processes,

• work organisation, and

• work conditions.

The key indicators are classified in different scales. The scales correspond to conditions in practice and range from a minimum/optimum to maximum/poor. The classification of these scales indicates potential bottlenecks for each category/indicator. By multiplying the scale value of the daily duration of activity with the sum of the other scale scores, a total value can be calculated. This calculated sum score can be used as a risk score. This score can be allocated to a risk range:

• „green": Low exposure situation, where physical overload is unlikely to occur.

• „greenish yellow": Increased exposure situation, physical overload is possible for less resilient subjects. Redesign of the workplace might be helpful for this group.

• „yellow": Highly increased exposure situation, physical overload also possible for normal subjects. Redesign of the workplace is recommended.

• „red": High exposure situation, physical overload is likely to occur. Redesign of the workplace is necessary.

### Samples

The study is carried out at different workplaces with different physical exposures to heavy forces, awkward postures, and repetitive manual handling operations. Groups of approximately 40 employees at each workplace are investigated. A minimum of 200 employees are considered in total.

A previous sample of office workers (684 men and 371 women, working at visual display units [5]) without relevant exposures from repetitive manual handling operations is used as reference group with regard to musculoskeletal symptoms.

### Power calculation

In a pilot study of machine operators (2 workplaces, 56 exposed subjects) the 12-month prevalence of symptoms in the elbow, hand/wrist and foot/ankle region was higher in female machine operators than in female controls in univariate analysis (51% vs. 16%; 55% vs. 25%; 31% vs. 11%). These results were significant in multivariate analyses (elbow region: OR 4.2 [95%-CI: 1.8 - 9.4]; hand/wrist region: OR 3.5 [95%-CI: 1.6 - 7.8]; foot/ankle region: OR 3.5 [95%-CI: 1.4 - 9.0]). The prevalence of symptoms in other body regions studied did not differ. Male machine operators more frequently reported symptoms in the hand/wrist region and knee region (12-month prevalence: 51% vs. 19% and 56% vs. 27%). These results were significant in multivariate analyses (hand/wrist region OR: 4.3 [95%-CI: 2.1 - 8.6]; knee region OR: 3.0 [95%-CI: 1.5 - 6.0]). In addition, a higher risk for foot/ankle symptoms (12-month prevalence) was calculated for exposed male workers (OR: 2.8 [95%-CI: 1.2 - 6.1]).

For power calculation EpiManager-Software was used [53]. Considering the results of this pilot study, the power of the study can be calculated as follows. Assuming only a small difference in the prevalences of symptoms between exposed and unexposed subjects of nearly 0.25 (55% - 30%, corresponding to a prevalence ratio of 1.83) the power (1-beta) of the study is calculated for men as 99% (n = 650 unexposed and n = 120 exposed men) and as 90% for women (n = 350 unexposed and n = 80 exposed women). If single workplaces with different exposures of manual handling operations are assessed, the number of exposed subjects is reduced to nearly 40 men or women per workplace. In this case and with regard to the conditions mentioned above, the power of the study is 86% (n = 40 exposed men) or 84% (n = 40 exposed women). The power calculation does not consider loss of power due to effects of confounders in the multivariate analysis.

### Analysis

Analysis of research topic 1: Assessing workplaces by means of the KIM-MHO, different occupational health and safety officers should obtain similar scores.

The objectivity is determined examining the independence of results assessed by different individuals. Descriptive statistics are used to show the distribution of different workplace assessments of the involved experts (mean, median, variance, range). Inter-rater reliability for multiple raters are analysed by using standard video sequences of typical workplaces for risk assessment and by rating these videos by a group of selected experts under standardised conditions [54].

Analysis of research topic 2: At the completion of the KIM-MHO, no significant descepancies occur between the opinions of the scientists and the operational workers involved.

At every workplace, an extensive work analysis is carried out to gather relevant data about respective manual handling operations (duration of tasks, frequency, force, posture, etc.). Based on this data, the KIM-MHO is then used by scientists and occupational health and safety officers seperately to assess the working conditions. The difference between the real exposure (as assessed by extensive work analysis) and the assessment by KIM-MHO will be used to describe the validity of the KIM-MHO. Descriptive statistics will be used to illustrate the distribution of different workplace assessments of the involved experts (mean, median, variance, range). If significant discrepancies occur, relevant parts of the KIM-MHO will be rephrased and adjusted. If rephrasing and adjusting is necessary, the modified KIM-MHO will be tested again for sufficient validity.

Analysis of research topic 3: It is assumed, that employees at workplaces with high exposures of manual handling operations show health related outcomes (musculoskeletal symptoms) more frequently than non-exposed workers, taking into consideration relevant confounders such as age, gender, constitution or disposition. Secondly it is assumed, that the KIM-MHO displays high scores at workplaces with high degrees of manual handling operations and high frequencies of musculoskeletal symptoms in exposed workers, and low scores at workplaces with low exposures of manual handling operations and low frequencies of musculoskeletal symptoms in workers.

To estimate whether prevalences of symptoms in the upper extremities and neck are excessive, the data from the employees of each workplace will be compared with a similar reference data set among employees working at visual display terminal (VDT) workstations. These reference data were generated in a cross-sectional study of 1,065 employees working at VDT [5]. In addition, the prevalence of the tentative diagnoses will be analysed to complement the data. The exposed and unexposed cohorts are described and compared in regard to different health related outcomes (descriptive statistics in regard to prevalence of symptoms in different parts of the body and other outcomes). Multivariate regression analysis based on log-binomial models will be used for multivariate comparisons between exposed and unexposed subjects. Prevalence Ratios will be calculated as effect estimates. Relevant confounders (age, constitution, disposition, behavior, work history) are taken into consideration. Directed Acyclic Graphs will be used in confounder selection [55]. Data will be generally stratified by gender. Higher prevalences of symptoms indicate that the KIM-MHO score should be high as well. If discrepancies occur between the prevalence of symptoms on one hand and the height of the KIM-MHO score on the other hand, the KIM-MHO will be adjusted. The association between the exposure to manual handling operations as assessed by means of the KIM-MHO and the frequency of musculoskeletal symptoms within the exposed workers will be determined (criterion validity). To assess the correlation between the KIM-MHO score and the prevalence of musculoskeletal symptoms, prevalence ratios are calculated (general linear model: log-binomial, adjusted for age, height and BMI, stratified for gender, 95% confidence intervals (95%-CI)).

### Quality control and assurance

The use of standardised and - if available and appropriate - already validated and/or evaluated instruments insures high quality of research. All questionnaires are completed during a face-to-face interview. The interviewer is the same for all workplaces. The physical examinations are performed by occupational physicians of the companies or external occupational health physicians from one of the participating research institutes. A standardised procedure for the physical examination is insured by specific standardised training of the examining physicians.

### Time frame of the study

The study team started with the planning of this project in summer 2008. The data collection then started in 2009 and ends in 2010. Description and analysis of the data will be done by the end of 2010. The approved or revised KIM-MHO will be presented publically in spring/summer 2011.

### Description of risks

To our knowledge, neither serious risks nor undesired effects can arise from completing the questionnaires or from the standardised physical examination by an occupational health physician. Nothing in regard to these effects has been reported in the literature. Thus, there seem to be no specific risks related to the study.

### Benefits of the study

This study will evaluate the new KIM-MHO for practical risk assessment in manual handling operations. In addition, the study will extend knowledge concerning the correlation between specific MSDs and characteristics of manual handling operations. The KIM-MHO is an important modular supplement to the practical methods assessing the risk factors associated with work-related musculoskeletal disorders.

### Ethical principles

The study was planned and conducted in accordance with the German medical professional code and the Helsinki Declaration of 1996 as well as the German Federal Data Protection Act. The study protocol and its amendment were approved by the Ethics Committee of the University of Witten/Herdecke (approval no. 35/2009). The study was started after the Ethics Committee gave its written and unrestricted approval.

Employees participate in the study voluntarily. They can end their participation at any time without reason and without negative consequences, e.g. for their job.

### Informed consent

Written informed consent for participation is obtained before the survey. Employees receive written and verbal information about the main features of the study as well as about potential benefits for their health and their contribution to the common public welfare. If they accept the conditions of the study and their participation, they document their consent with their signature. A copy of this statement is intended to be kept by the employee for later reference or cancellation of participation. In the event of study discontinuation, all data will be deleted, unless the employee explicitly wishes and affirms further analysis of his/her data.

### Data security/disclosure of original documents

All original documents are treated in accordance with the German Federal Data Protection Act. All study-related data and documents are stored on a secured central server of the study centre. Only selected members of the study team have access to the respective files.

## Discussion

This research project will provide a scientific substantiation and, if necessary, modification of a new method to assess manual handling operations at workplaces. In addition, the knowledge about the correlation between work-related factors and different musculoskeletal disorders in the upper limb region will be expanded. With this knowledge, a better classification of occupational hazards with regard to musculoskelettal upper limb diseases will be available in future. This might lead to more specific prevention strategies.

## Abbreviations

BAUA: Bundesanstalt für Arbeitsschutz und Arbeitsmedizin [Federal Institute for Occupational Safety and Health] in Germany; COPSOQ: Copenhagen Psychosocial Questionnaire; EEC: European Council Directive; FEBA: Fragebogen zur subjektiven Einschätzung der Belastungen am Arbeitsplatz [Questionnaire of the subjective estimation of exposures]; KIM: Leitmerkmalmethode [Key Indicator Method]; LASI: Landerausschuss für Arbeitsschutz und Sicherheitstechnik [Committee of the German states for Occupational Safety and Health] in Germany; LHC: Lifting/Holding/Carrying; MHO: Manual Handling Operations; MSD: Musculoskeletal Disorders; PP: Pulling/Pushing; UEMSDS: Upper Extremity Muskulo-Skeletal Disorders; VDT: Visual Display Terminal

## Competing interests

The authors declare that they have no competing interests.

## Authors' contributions

All authors conceived and designed the study and prepared this manuskript. US and FL represent the funding body. US developed the draft of the KIM and initiated this study. Both were closely involved in the planning and development of the study design and preparing the study protocol. All authors read and approved the final manuscript.

## Pre-publication history

The pre-publication history for this paper can be accessed here:

http://www.biomedcentral.com/1471-2474/11/272/prepub
